# Preoperative FluoroCT imaging -guided transapical closure of mitral paravalvular leak: a case report

**DOI:** 10.3389/fcvm.2026.1764024

**Published:** 2026-03-19

**Authors:** Qiong Luo, Ya Li, Hongkun Wu, Hao Chen

**Affiliations:** 1 Department of Cardiology, Chongqing General Hospital, Chongqing University, Chongqing, China; 2 Department of Cardiovascular Surgery, Chongqing General Hospital, Chongqing University, Chongqing, China

**Keywords:** closure of mitral paravalvular leak, FluoroCT imaging -guided, minimally invasive, precise preoperative positioning, transapical

## Abstract

**Background:**

Mitral paravalvular leak (PVL) is a recognized complication following prosthetic valve replacement, associated with heart failure, hemolysis, and increased morbidity. While redo surgery is the traditional treatment, transcatheter closure has emerged as a less invasive alternative, particularly in high-risk surgical candidates. Here, we present a case of preoperative FluoroCT imaging planning for transapical closure of mitral PVL.

**Case presentation:**

A 57-year-old female with prior double mechanical valve replacement (mitral and aortic) presented with worsening exertional dyspnea and fatigue.Transesophageal Echocardiography (TEE) identified a 5 mm posterior paravalvular leak adjacent to the mitral sewing ring. Preoperative contrast-enhanced CT analyzed with FluoroCT imaging software localized the leak to the 4–5 o'clock position and provided the optimal C-arm projection (cranial 48°, left anterior oblique 8°). Transapical closure via a left mini-thoracotomy was performed, and a 10 mm Ventricular Septal Defect occluder (Huayishengjie Medical Company, Beijing, China) was successfully deployed under TEE and fluoroscopic guidance. The patient had no residual leak, and total procedure time was 1 hour.

**Conclusions:**

FluroCT imaging-guided preoperative CT planning enables accurate localization of PVL and optimal fluoroscopic projection, reducing procedure time and improving safety. Transapical closure remains a feasible and effective approach for posterior mitral PVL in complex double-valve patients.

## Background

Paravalvular leak (PVL) is a relatively common complication following prosthetic mitral valve replacement, with echocardiographic incidence ranging from 7% to 17% (1). While many PVLs are clinically silent, approximately 1%–5% are clinically significant, leading to heart failure, hemolysis, or increased mortality (2, 3). Redo open surgery remains the traditional gold standard but carries substantial risk, particularly in reoperative settings. In recent years, transcatheter PVL closure has become an important alternative, with high technical success rates and improved patient outcomes (4–6) P.Pre-procedural imaging modalities including 3D echocardiography, MDCT, and advanced planning software play a critical role in diagnosis, prosthetic valve evaluation, pre-procedural planning, and intraprocedural guidance (7–9). FluoroCT imaging software is a novel tool that enhances preoperative planning by simulating x-ray projections to minimize interference from prosthetic components.

## Case presentation

**Figure 1:**
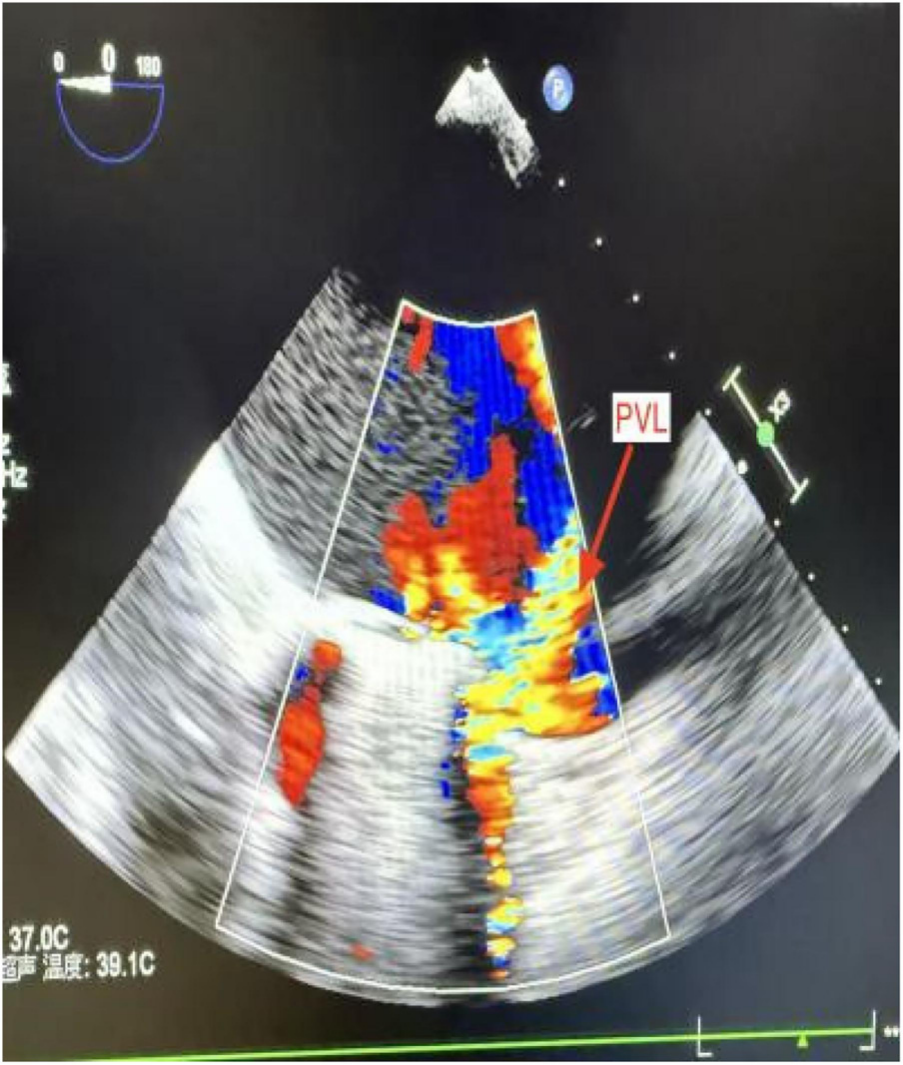
Transesophageal echocardiography (TEE) demonstrated a 5 mm PVL adjacent to the posterior mitral annulus.

**Figure 2:**
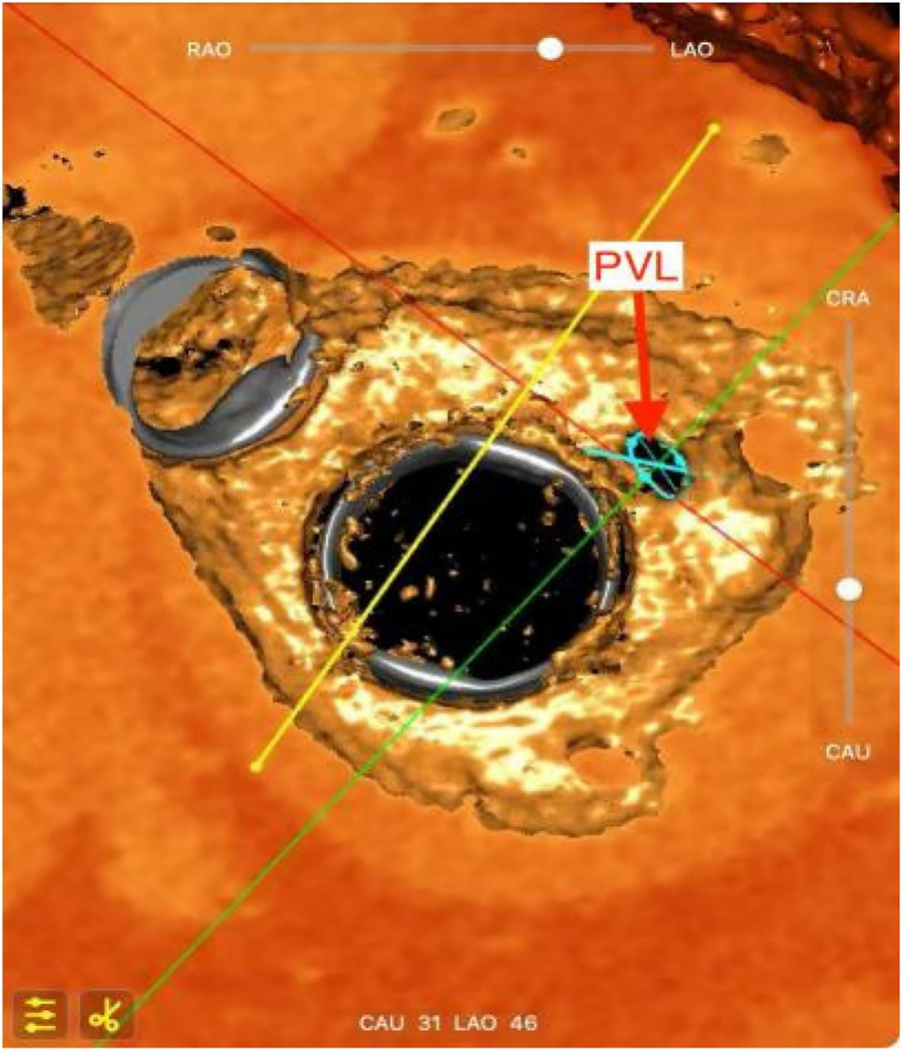
Preoperative CT analyzed using FluoroCT imaging software localized the PVL at the 4–5 o’clock position.

**Figure 3:**
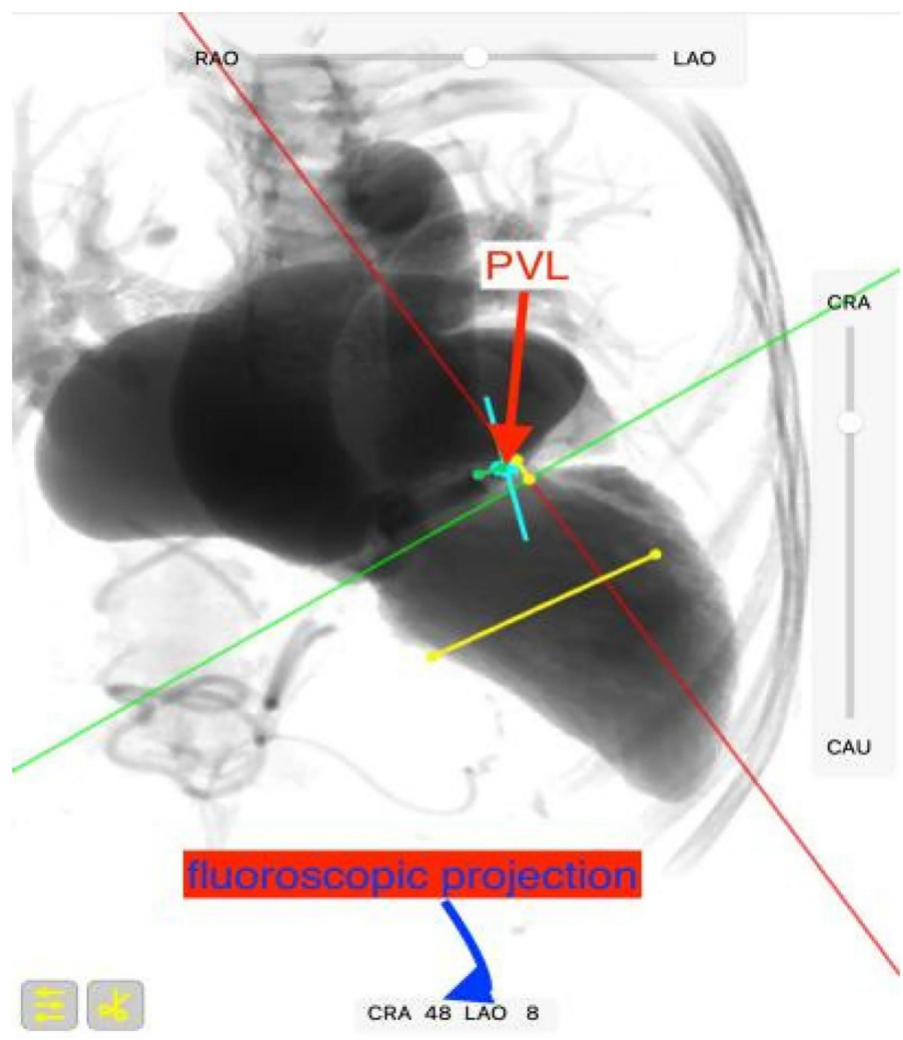
Preoperative CT analyzed using FluroCT identified the optimal fluoroscopic projection (CRA 48°, LAO 8°) to minimize interference from prosthetic components.

**Figure 4:**
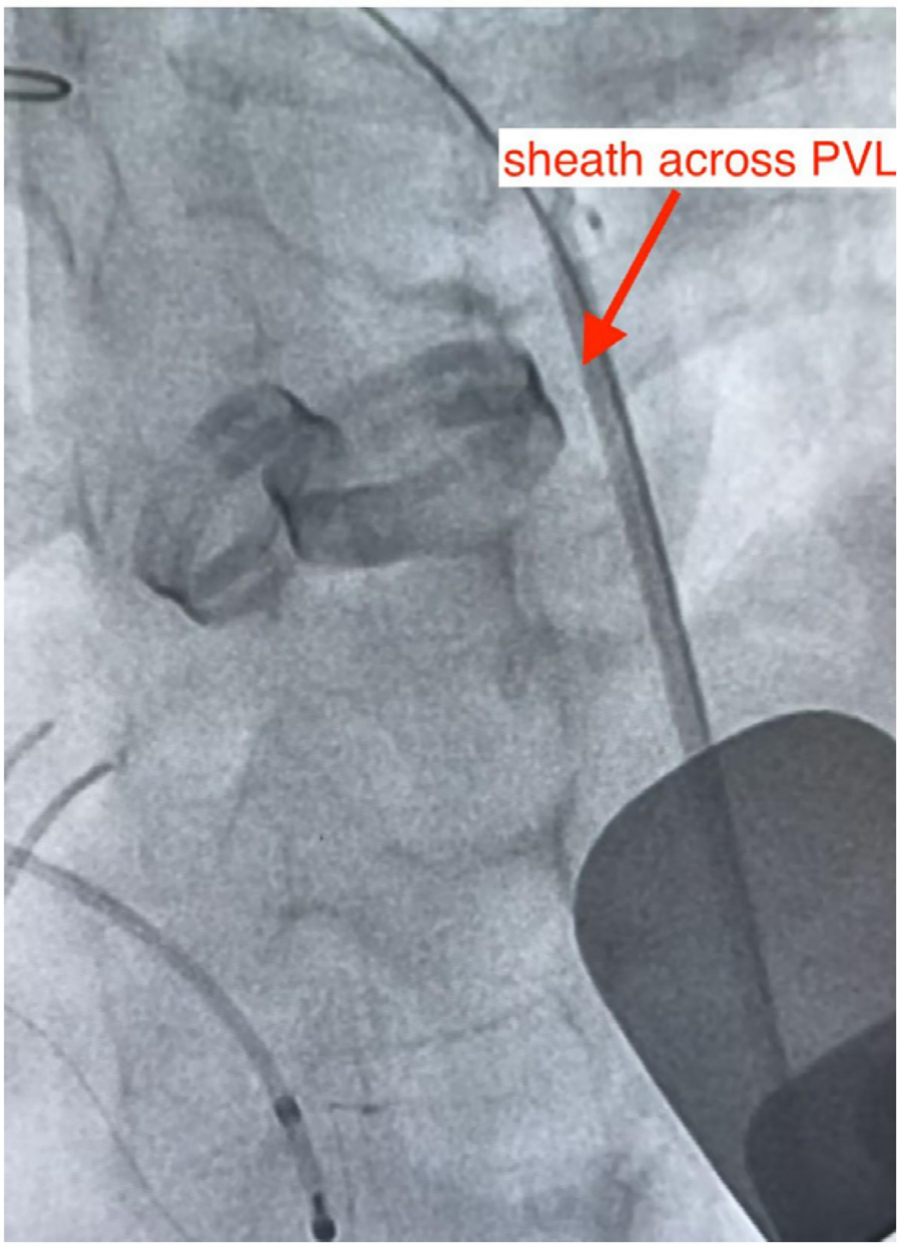
According to the preoperative plan, the optimal fluoroscopic projection angle (CRA 48°, LAO 8°) on DSA allowed easy intraoperative localization of the PVL, the defect was successfully crossed with a guidewire and catheter, thereby establishing a stable delivery tract for device deployment.

**Figure 5:**
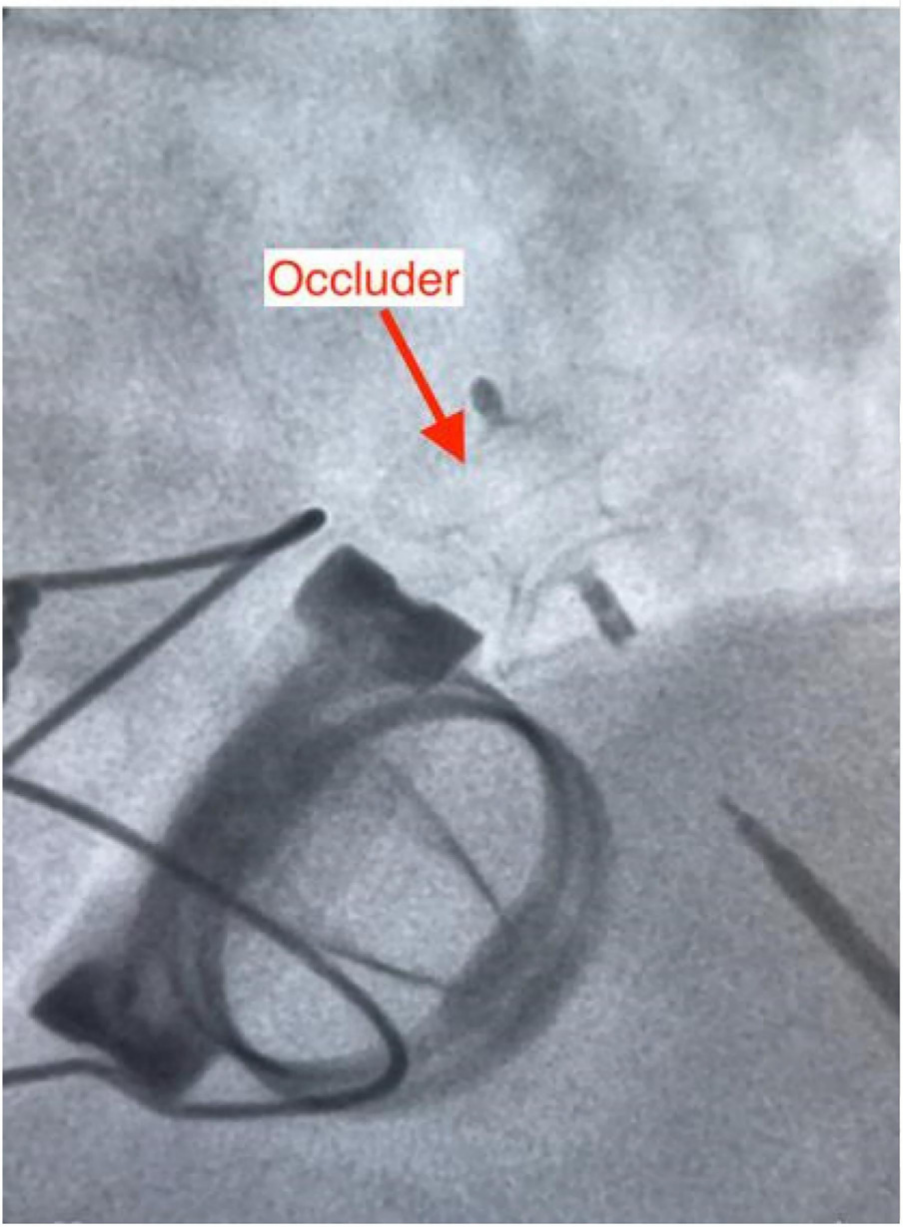
A 10 mm ventricular septal defect occluder was deployed with proper seating.

**Figure 6:**
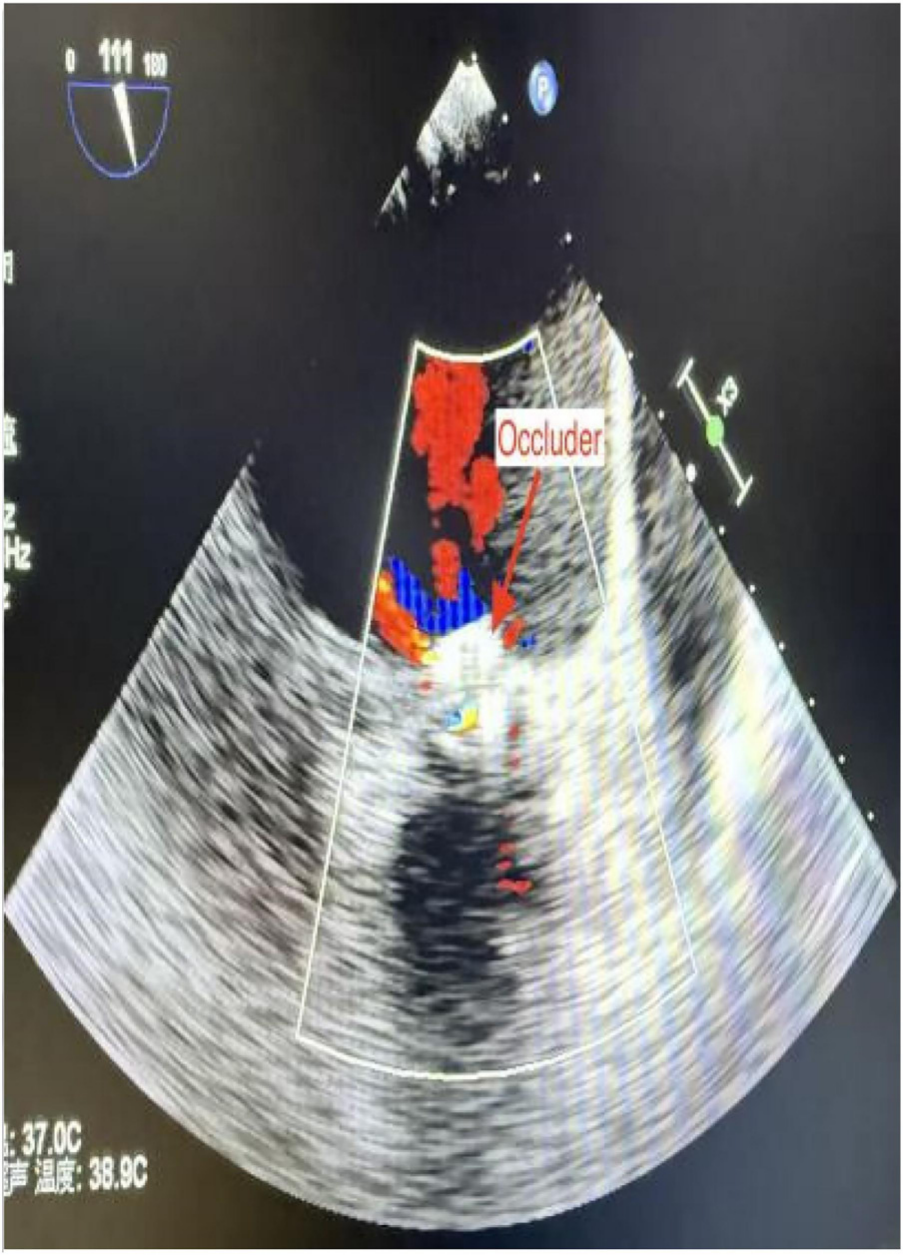
Postoperative TEE confirmed no residual leak.

A 57-year-old woman with a history of double mechanical valve replacement (mitral and aortic, 5 years prior) presented with a 3-month history of worsening exertional dyspnea and fatigue. There were no clinical signs or symptoms of hemolysis in this patient.Physical examination revealed a new systolic murmur. Transesophageal Echocardiography (TEE) demonstrated a 5 mm PVL adjacent to the posterior mitral annulus (Figure 1), with left ventricular dilatation (LVEDD 57 mm, LVESD 46 mm) and mildly reduced ejection fraction (46%). Preoperative CT analyzed using FluoroCT imaging software (version 3.2, Circle Cardiovascular Imaging Inc. Calgary,Canada) localized the PVL at the 4–5 o'clock position (Figure 2) and identified the optimal fluoroscopic projection (CRA 48°, LAO 8°) to reduce interference with prosthetic valve (Figure 3). Given the patient's mechanical aortic valve prosthesis, a transaortic approach for mitral paravalvular leak closure was not feasible. Although a transseptal approach was considered.given the lateral/posterior location of the leak, we ultimately selected a transapical route to maximize coaxiality and catheter support. In this double-mechanical- valve patient, extensive catheter manipulation within the left atrium may increase the risk of prosthesis interference. Moreover, the posterior 4–5 o'clock PVL location often requires significant catheter deflection from a transseptal trajectory, potentially resulting in suboptimal alignment and unstable crossing. Finally, prior transseptal surgery with an interatrial septal patch/scar may increase uncertainty of puncture site and resistance during sheath passage, thereby prolonging the procedure and increasing the risk of iatrogenic septal injury. Given these considerations, transapical access provided a shorter, more coaxial and controlled path for device delivery. Under general anesthesia, a left mini-thoracotomy at the fifth intercostal space was performed to expose the Left Ventricle (LV) apex. After purse-string placement, A 7F delivery sheath was advanced via a guidewire across the defect under fluoroscopy(C-arm angulations CRA 48°, LAO 8°) (Figure 4). A 10 mm Ventricular Septal Defect Occluder (waist diameter 10 mm, waist height 6 mm, and disc diameters 14 mm on both the left and right sides) (Huayishengjie Medical Company, Beijing, China) was deployed with proper seating, avoiding interference with prosthetic leaflets (Figure 5). TEE confirmed no residual leak (Figure 6). The apex was closed securely, and the total procedure lasted approximately 60 min.

## Discussion

Mitral PVL remains a challenging complication of prosthetic valve surgery. Significant leaks contribute to increased morbidity and mortality (10, 11). Redo surgery, while effective, is associated with high risk in reoperative patient (12). Transcatheter closure has show success rates exceeding 80%–90% in recent multicenter series (13, 14). The choice of access route depends largely on leak location. Posterior leaks, as in this case, are often more accessible via the transapical route, which provides a direct and coaxial trajectory (15).

Advanced imaging is crucial in procedural planning. 3D TEE provides real-time anatomical guidance (16), while CT enables precise localization and device sizing (17). In our case, preoperative FluoroCT imaging software adds value by predicting optimal C-arm angulations to reduce interference with prosthetic valve, thereby facilitating efficient leak crossing and device deployment, reducing procedure time and improving safety.

In our workflow, contrast-enhanced CT served as the primary modality for anatomic characterization and procedural planning, including PVL localization and prediction of the optimal fluoroscopic projection using FluoroCT simulation. Fluoroscopy remained essential for real-time tracking of wires, catheters and device deployment as well as immediate assessment of potential mechanical leaflet impingement. TEE (preferably 3D TEE) provided complementary real-time evaluation of PVL morphology, device–prosthesis interaction, and residual regurgitation. This report highlights the integration of novel imaging tools with established surgical- transcatheter hybrid approaches. Future studies in larger patient cohorts are warranted to validate the reproducibility, safety, and long-term outcomes of FluoroCT imaging -guided PVL closure.

## Conclusions

Preoperative FluoroCT imaging software -assisted CT analysis can accurately localize mitral PVLs, optimize fluoroscopic angulation, and facilitate efficient transapical closure in complex double-valve patients. This approach may reduce procedure time, radiation exposure, and procedural complications, representing a promising adjunct in structural heart interventions.

## Data Availability

The original contributions presented in the study are included in the article/Supplementary Material, further inquiries can be directed to the corresponding author.
